# Annual acknowledgement of manuscript reviewers

**DOI:** 10.1186/1478-4491-12-4

**Published:** 2014-01-31

**Authors:** Mario Roberto Dal Poz

**Affiliations:** 1 Human Resources for Health, University of the State of Rio de Janeiro, Rio de Janeiro, Brazil

## Abstract

**Contributing reviewers:**

The editors of *Human Resources for Health* would like to thank all our reviewers who have contributed to the journal in Volume 11 (2013).

## 

Olaf Aasland

Norway

Orvill Adams

Serbia

Adam Ahmat

Congo

Ezaz Ahmed

Australia

Emily Ahonen

United States of America

Alvaro Alonso-Garbayo

Mozambique

Woldekidan Kifle Amde

Ethiopia

Edson Araujo

United States of America

Rafael Arouca

Brazil

Juan Arroyo

Peru

Rebecca Bailey

United States of America

Jane Ball

United Kingdom

Lesley Barclay

Australia

Jean Barry

Switzerland

Nicola Barsdorf

South Africa

Ken Bassett

Canada

Kisalaya Basu

Canada

Ronald Batenburg

Netherlands

Samantha Battams

Switzerland

Andrea Baumann

Canada

Elizabeth Beddoe

New Zealand

Linda Benskin

United States of America

Anita Bercovitz

United States of America

Trine Strand Bergmo

Norway

Posy Bidwell

United Kingdom

Judy Bloom

Canada

Ana Luiza Borges

Brazil

Peter Brooks

Australia

James Buchan

Australia

Jim Campbell

Spain

Pietro Canepari

Italy

Christopher Carroll

United Kingdom

Cristiana Carvalho

Brazil

Janete Castro

Brazil

Helen Cleak

Australia

Jeremye Cohen

United States of America

Christopher Colvin

South Africa

John Connell

Australia

Karen Cook

Australia

Adrijana Corluka

United States of America

Isabel Correia

Portugal

Ian Couper

South Africa

Isabel Craveiro

Portugal

Fernando Cupertino De Barros

Brazil

Elina Dale

Norway

Brian Dangerfield

United Kingdom

Karen Daniels

South Africa

Anita Alero Davies

Switzerland

Aileen Davis

Canada

Angela Dawson

Australia

Margaret Dawson

Australia

Frances Day-Stirk

United Kingdom

Kenneth de Camargo

Brazil

Korrie de Koning

Netherlands

Jan De Maeseneer

Belgium

Paolo De Paoli

Italy

Marietjie De Villiers

South Africa

Dianne Delva

Canada

Rachel Deussom

United States of America

Emmanuel d'Harcourt

United States of America

Marjolein Dieleman

Netherlands

Mamuka Djibuti

Georgia

Carole Doherty

United Kingdom

Carmen Dolea

Switzerland

Delanyo Dovlo

Switzerland

Norbert Dreesch

Austria

Christine Duffield

Australia

Gilles Dussault

Portugal

Kenneth Eaton

United Kingdom

Elizabeth Ekirapa-Kiracho

Uganda

Nazar Elfaki

Sudan

Carol Hall Ellenbecker

United States of America

Rene English

South Africa

Catrin Evans

United Kingdom

Emily Evens

United States of America

Fleming Fallon

United States of America

Victoria Fan

United States of America

Jane Farmer

Australia

Frank Feeley

United States of America

Paulo Ferrinho

Portugal

Laura Feuerwerker

Brazil

Kevin Fiscella

United States of America

Karin Fisher

Australia

Yolanda Flores-Peña

Mexico

Pierre Fournier

Canada

Seble Frehywot

United States of America

Christopher Friese

United States of America

Ines Fronteira

Portugal

Anastasia Gage

United States of America

Omar Galarraga

United States of America

Jenny Gallagher

United Kingdom

Moses Galukande

Uganda

Brenda Gamble

Canada

Pape Gaye

United States of America

Gulin Gedik

Switzerland

Asha George

United States of America

Barend Gerretsen

Myanmar

Basu Ghosh

Oman

Marisa Gilles

Australia

Irene Glinos

Belgium

Beatriz González López-Valcárcel

Spain

Saji Gopalan

United States of America

Des Gorman

New Zealand

Jeff Gow

Australia

Neeru Gupta

Canada

Omer Guzel

Turkey

Ana Estela Haddad

Brazil

Sharon Hakkennes

Australia

Thomas Hall

United States of America

Christine Hancock

United States of America

Paula Hancock

United Kingdom

Mark Hann

United Kingdom

Claudia Hanson

Sweden

Piya Hanvoravongchai

Thailand

Nida Harahap

Indonesia

Bernice Harris

South Africa

Richard Hays

Australia

Sibasis Hense

Australia

Patricia Hernandez

Switzerland

Helena Hildenwall

Sweden

David Hipgrave

Australia

Miriam Hirschfeld

Israel

Sara Holmes

United Kingdom

Caroline Homer

Australia

Charles Hongoro

South Africa

Jannie Hugo

South Africa

John Humphreys

Australia

Niamh Humphries

Ireland

Steinar Hunskaar

Norway

Samia Hurst

Switzerland

Hiroo Ide

Japan

Maia Ingram

United States of America

Natalie Jackson

New Zealand

Diana Jamal

Lebanon

Edgar Jarillo

Mexico

Wanda Jaskiewicz

United States of America

Masamine Jimba

Japan

Pongpisut Jongudomsuk

Thailand

Karien Jooste

South Africa

Lukas Kaelin

Austria

Jonathan Kagan

United States of America

Per Kallestrup

Denmark

Terhi Kankaanranta

Finland

Sheila Keane

Australia

Mark Keim

United States of America

Hafiz Khan

United Kingdom

Naresh Khatri

United States of America

Karina Kielmann

United Kingdom

Juliet Kiguli

Uganda

Jillian Clare Kohler

Canada

Maryse Kok

Netherlands

Joseph Kolars

United States of America

John Kolbe

New Zealand

Riitta-Liisa Kolehmainen-Aitken

Spain

Daniela Koller

Germany

Ellen Kuhlmann

Germany

Saravana Kumar

Australia

Susanne Kvarnström

Sweden

Sarah Larkins

Australia

Rene Lavallee

Canada

Deborah Law

Australia

How Lee

Canada

Sandra Leggat

Australia

Michael Leiter

Canada

Christophe Lemiere

United States of America

Juan Alfonso Leonardia

Philippines

Tomas Lievens

United Kingdom

Rosemary Lim

United Kingdom

João Fernando Lima Schwalbach

Mozambique

Vivian Lin

Australia

Lisa Little

Canada

George Liu

Australia

Zhaorui Liu

China

Lesley-Anne Long

United Kingdom

Jordan Louviere

Australia

Cid Manso de Mello Vianna

Brazil

Martha Macleod

Canada

Agya Mahat

United States of America

Claudia Maier

Germany

Mats Malqvist

Sweden

Ogenna Manafa

Ireland

Rachel Manongi

Tanzania

Annick Manuel

Switzerland

Paul Marschall

Germany

Tim Martineau

United Kingdom

José Martins

Portugal

Keith Masnick

Australia

Wanjiku Mathenge

Rwanda

Maria Mathews

Canada

Masatoshi Matsumoto

Japan

Patrick Mbindyo

Kenya

Eilish McAuliffe

Ireland

James McCaffery

United States of America

Mark McCarthy

United Kingdom

Gordon McCord

United States of America

David McCoy

United Kingdom

Matthew McGrail

Australia

Matthew McHugh

United States of America

Barbara McPake

United Kingdom

Lori Melichar Gadkari

United States of America

Hugo Mercer

Argentina

Anthony Montgomery

Greece

Jean Moore

United States of America

Fernando Mora-Carrasco

Mexico

Emmanuel Mpinga Kabengele

Switzerland

Malay Mridha

Bangladesh

Kamolnud Muangyim

United States of America

Luke Mullany

United States of America

Adiel Mushi

Tanzania

Helen Nabwera

United Kingdom

Lucio Naccarella

Australia

Paulo Nadanovsky

Brazil

Susan Nancarrow

Australia

David Nash

United States of America

Eva Naznin

Bangladesh

Saharnaz Nedjat

Iran

Joel Negin

Australia

Lisa Nelson

Switzerland

Peter Ngatia

Kenya

Gustavo Nigenda

Mexico

Mwansa Annette Nkowane

Switzerland

Mirko Noordegraaf

Netherlands

Ezekiel Nukuro

Fiji

Jennifer Nyoni

Congo, Democratic Republic

Jacinta Nzinga

Kenya

Edward Okeke

United States of America

Kimberly Oman

Australia

Mary O'Neil

United States of America

Tomoko Ono

France

Gorik Ooms

Belgium

Emmanuel Otolorin

Nigeria

Judith Oulton

Switzerland

Nonglak Pagaiya

Thailand

Barbara Parfitt

United Kingdom

Sarah Parker

United States of America

Edith Patouillard

United Kingdom

Marina Peduzzi

Brazil

James Pfeiffer

United States of America

David Phillips

United States of America

Siriwan Pitayarangsarit

Thailand

Karen Plager

United States of America

Krit Pongpirul

Thailand

Surabhi Purohit

India

Estelle Quain

United States of America

Magdalena Raban

Australia

Howard Rabinowitz

United States of America

Stephen Reid

South Africa

Wolfgang Rennert

United States of America

Thomas Ricketts

United States of America

Andrew Ries

United States of America

Patricia Riley

United States of America

Laetitia Rispel

South Africa

Narjis Rizvi

Pakistan

Graham Roberts

Australia

Dilys Robinson

United Kingdom

Jean Robson

United Kingdom

Paul Robyn

United States of America

Bernadette Rodak

United States of America

Janetta Roos

South Africa

Samantha Rowe

United States of America

Giuliano Russo

Portugal

Mandy Ryan

United Kingdom

Gerard Schmets

Switzerland

Deborah Schofield

Australia

Adrian Schoo

Australia

Michael Schriver

Denmark

Anthony Scott

Australia

Juan Scribante

South Africa

La Rue Seims

United States of America

Tarun Sen Gupta

Australia

Sukyong (Sue) Seo

Korea, South

Ilaria Setti

Italy

Dykki Settle

United States of America

Dale Sheehan

New Zealand

Kenneth Sherr

United States of America

Mohsin Sidat

Mozambique

Mariangela Simao

Switzerland

Prabhjot Singh

United States of America

Amani Siyam

Switzerland

Brigita Skela Savic

Slovenia

Marc Soethout

Netherlands

Allison Squires

United States of America

Michael Stalker

United States of America

Barbara Stilwell

United States of America

Laura Stormont

Switzerland

Roger Strasser

Canada

Sakari Bertel Alfred Suominen

Finland

Jamsheer Talati

Pakistan

Shinichi Tanihara

Japan

Douglas Taren

United States of America

Christine Tashobya

Uganda

Hailay Teklehaimanot

Ethiopia

Sally Theobald

United Kingdom

Steve Thomas

Ireland

Kea Tijdens

Netherlands

Shin-ichi Toyabe

Japan

Peter Trewby

United Kingdom

Carolyn Tuohy

Canada

Kerry Uebel

South Africa

Kim Jocelyn Usher

Australia

Frédérique Vallières

Ireland

Iat Kio Van

Macao

Ankie A.L.J. van den Broek

Netherlands

Joost van der Gulden

Netherlands

Malou Van Greuningen

Netherlands

Wim Van Lerberghe

Portugal

Sarah Vickerstaff

United Kingdom

Dilys Walker

United States of America

P.J. Wall

Ireland

Hideomi Watanabe

Japan

Heide Weishaar

United Kingdom

Jennifer Mary Weller

New Zealand

Stuart Whittaker

South Africa

Tim Wilkinson

New Zealand

Matthias Wismar

Belgium

Kara Wools-Kaloustian

United States of America

Ying Xue

United States of America

Débora Ingrid Yanco

Argentina

Zeki Yildiz

Turkey

Junhua Zhang

China

Pascal Zurn

Switzerland

Prisca Zwanikken

Netherlands

